# Molecular typing of multidrug-resistant Salmonella Blockley outbreak isolates from Greece.

**DOI:** 10.3201/eid0601.000111

**Published:** 2000

**Authors:** P T Tassios, C Chadjichristodoulou, M Lambiri, A Kansouzidou-Kanakoudi, Z Sarandopoulou, J Kourea-Kremastinou, L S Tzouvelekis, N J Legakis

**Affiliations:** University of Athens, Greece.

## Abstract

During 1998, a marked increase (35 cases) in human gastroenteritis due to Salmonella Blockley, a serotype rarely isolated from humans in the Western Hemisphere, was noted in Greece. The two dominant multidrug-resistance phenotypes (23 of the 29 isolates studied) were associated with two distinct DNA fingerprints, obtained by pulsed-field gel electrophoresis of genomic DNA.


### Molecular Typing of Multidrug-Resistant *Salmonella* Blockley Outbreak Isolates from Greece

**Panayotis T. Tassios,* Christos Chadjichristodoulou,† Maria Lambiri,‡ Athina Kansouzidou-Kanakoudi,§ Zannina Sarandopoulou,* Jenny Kourea-Kremastinou,‡ Leonidas S. Tzouvelekis,* Nicholas J. Legakis***
*University of Athens, Athens, Greece; †National Center for Surveillance and Intervention, Athens, Greece; ‡National School of Public Health, Athens, Greece; and §Salmonella Reference Center for Macedonia and Thrace, Thessaloniki, Greece

During 1998, a marked increase (35 cases) in human gastroenteritis due to *Salmonella* Blockley, a serotype rarely isolated from humans in the Western Hemisphere, was noted in Greece. The two dominant multidrug-resistance phenotypes (23 of the 29 isolates studied) were associated with two distinct DNA fingerprints, obtained by pulsed-field gel electrophoresis of genomic DNA.

*Salmonella* Blockley is rarely isolated in the Western Hemisphere. According to Enter-net, the international network for surveillance of *Salmonella* and verocytotoxin-producing *Escherichia coli* infections, *S.* Blockley represented 0.6% of all *Salmonella* serotypes isolated in Europe during the first quarter of 1998, a full 100-fold lower than the dominant serotype, *S.* Enteritidis (67.1%) (1). However, *S.* Blockley is among the five most frequently isolated serotypes from both avian and human sources in Japan (2,3), Malaysia (4,5), and Thailand (6). A single foodborne outbreak in the United States (7) and sporadic human infections in Europe associated with travel to the Far East (8), animal infection (9) or carriage (10,11), and environmental isolates have also been reported (12,13).

Regardless of the frequency of *S.* Blockley isolation, its rates of resistance to antibiotics have been high. Among Spanish salmonellae isolated from natural water reservoirs, *S.* Blockley and *S.* Typhimurium had the highest rates of multidrug resistance (12). Comparing 1980-1989 with 1990-1994, researchers from Tokyo noted an increase in the number of *S.* Blockley isolates resistant to one or more antibiotics, from 92.0% to 98.2% for imported cases and from 57.4% to 88.7% for domestic cases (3,14). In Thailand, isolates from human or other sources also had high rates of resistance to streptomycin, tetracycline, kanamycin, and chloramphenicol and lower rates to ampicillin and trimethoprim/sulfamethoxazole (15).

Address for correspondence: Nicholas J. Legakis, Department of Microbiology, Medical School, University of Athens, M. Asias 75, 115 27 Athens, Greece; fax: 301-7709-180; e-mail: nlegakis@cc.uoa.gr.

Nevertheless, few attempts at typing *S.* Blockley isolates with molecular methods have been described, and these have been limited to the characterization of plasmid content (2,16).

During the second and third quarters of 1998, Enter-net reported higher numbers of *S.* Blockley isolates than during the same period of the previous year in several European countries (1). The epidemiologic investigations conducted in Germany, England and Wales, and Greece did not confirm a source for this increase (17-19).

In this study, we characterized the Greek outbreak isolates further, both with respect to their antibiotic resistance phenotypes and DNA fingerprints obtained by pulsed-field gel electrophoresis of genomic DNA.

### The Study

The study sample consisted of 28 of 35 *S.* Blockley strains isolated from May to December 1998 (19), one strain from February 1999, and four epidemiologically unrelated control strains: one from 1996 and three from 1997. All isolates were from human cases of enteritis. Identification was performed by the API 20E system (BioMerieux S.A., Marcy l'Etoile, France) and serotyping with commercially obtained antisera (BioMerieux) (20).

Susceptibility to kanamycin, streptomycin, ampicillin, amoxicillin/clavulanic acid, cefepime, tetracycline, chloramphenicol, trimethoprim/sulfamethoxazole, gentamicin, nalidixic acid, and ciprofloxacin was tested by a disk diffusion assay according to National Committee for Clinical Laboratory Standards guidelines (21). Genomic DNA was prepared and digested with *Xba*I (New England Biolabs) (22). Chi-square tests or Fisher exact tests were used to calculate two-tailed probabilities.

*S.* Blockley accounted for seven of the 13,199 salmonella isolates identified in Greece from 1976 to 1997. However, 35 gastroenteritis cases due to this serotype were reported from May to December 1998 (19). Twenty-nine *S.* Blockley strains isolated from fecal specimens of patients with gastroenteritis during May 1998 to February 1999, along with four epidemiologically unrelated clinical isolates from 1996 and 1997, were therefore studied for susceptibility to antibiotics. The 1998 outbreak isolates were scattered throughout Greece; *S.* Blockley was isolated later, starting in August 1998, in northern Greece.

All isolates were susceptible to trimethoprim/sulfamethoxazole, ampicillin, amoxicillin/clavulanic acid, gentamicin, and ciprofloxacin (Table). High resistance rates were observed to tetracycline (100%), streptomycin and kanamycin (90%), chloramphenicol (83%), and nalidixic acid (52%). Six resistance phenotypes could be distinguished (Table) with the two major phenotypes of outbreak isolates being resistant to kanamycin, streptomycin, tetracycline, and chloramphenicol (ATC) or kanamycin, streptomycin, tetracycline, chloramphenicol, and nalidixic acid (ATCN). Most (76%) strains isolated after August 24, 1998, were nalidixic acid-resistant (resistance phenotypes ATCN, TCN, ATN), unlike strains isolated up to August 17, 1998 (17%) ($1.29 < RR = 3.03 < 7.11$, $p = 0.005$).

When pulsed-field gel electrophoresis was used to obtain DNA fingerprints for these isolates (Figure 1), all belonged to the same type, A, although eight subtypes, A1-A8, could be distinguished on the basis of one to three DNA

Table. Resistance phenotypes for isolates of *Salmonella* Blockley in Greece

| PFGE[a] subtype (n) | Resistance phenotype[b] (n) | Time span | Locations[c] |
|---|---|---|---|
| A1 (1) | AT (1) | May 3, 1998 | 1 |
| A2 (12) | ATC (9), TCN (1), AN (1), AT (1) | 1997; May 16-Oct 13, 1998 | 6 |
| A3 (3) | ATC (1), ATN (1), TCN (1) | Aug 24-Oct 27, 1998 | 3 |
| A4 (12) | ATCN (8), ATN (2), ATC (1), TCN (1) | Aug 17, 1998-Feb 23, 1999 | 6 |
| A5 (1) | ATCN (1) | Sep 29, 1998 | 1 |
| A6 (1) | AT (1) | 1996 | - |
| A7 (1) | ATCN (1) | 1997 | - |
| A8 (2) | ATC (1), AN (1) | 1997; Aug 10, 1998 | 1 |

[a]PFGE, pulsed-field gel electrophoresis.
[b]A, kanamycin and streptomycin; T, tetracycline; C, chloramphenicol; N, nalidixic acid.
[c]Number of locations of isolation during the outbreak.

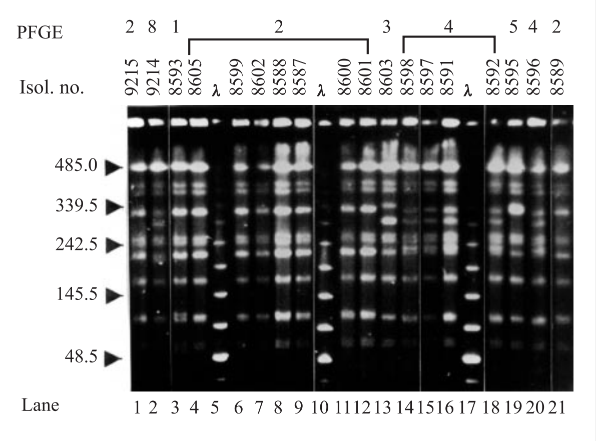

Figure 1. Sample pulsed-field gel electrophoresis (PFGE) gel of representative *Salmonella* Blockley isolates, indicating common and unique DNA fingerprints. Electrophoresis was through 1% agarose/0.5 x TBE, in a CHEF DRIII apparatus (BioRad Laboratories), at 14°C with a 120° switch angle and a run time of 20 hours, with a linear ramp of switching times from 5 to 32 seconds. Gels were stained with 0.5 mg/L ethidium bromide and documented under UV illumination by the EasyWin32 system (HeroLab, Germany). Images were assessed visually, and different PFGE subtypes (A1, A2, ...) were assigned to isolates with electrophoretic patterns differing by one to three DNA fragments (23). Gel images were also processed by the GelCompar software (Applied Maths, Kortrijk, Belgium), and on the basis of PFGE pattern similarities, a dendrogram was constructed by using the Dice coefficient and clustering by the unweighted pair group method, which uses arithmetic averages (UPGMA) with a 2% tolerance in band position difference.

Isol. no.: isolate number. PFGE: subtypes of PFGE type A. The sizes, in kb, of lambda phage DNA concatamers (New England Biolabs) are shown to the left of the gel. All lanes are from the same gel.

fragment differences (Figures 1, 2). Two of the four isolates from previous years belonged to unique subtypes A6 and A7; the other two belonged to subtypes A2 and A8, shared by outbreak isolates (Table). In contrast, 93% of the 1998 outbreak strains yielded PFGE patterns common to two or more isolates. Indeed, most outbreak isolates were grouped in subtypes A2 and A4, consisting of 11 and 12 isolates, respectively (Table).

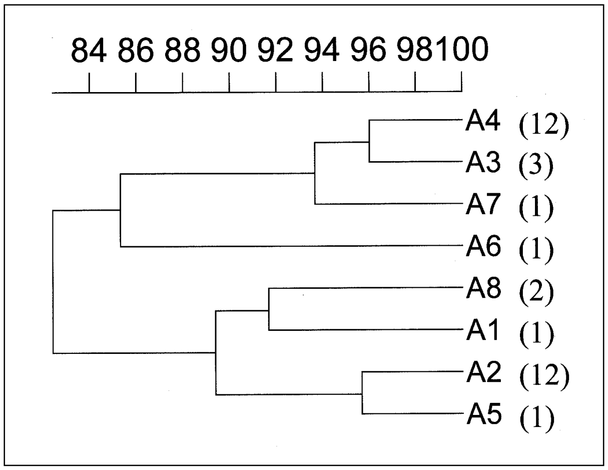

Figure 2. Dendrogram of similarity among the observed pulsed-field gel electrophoresis patterns. A percentage scale of similarity is indicated at the top. Numbers in parentheses refer to the number of isolates with the indicated pulsed-field gel electrophoresis pattern.

Pulsed-field gel electrophoresis subtypes were associated with resistance phenotypes. Most resistance phenotype ATC isolates belonged to subtype A2 (3.03. <RR = 11.86 <46.5, p = 0.0000098), while most resistance phenotype ATCN isolates belonged to A4 (2.76 <RR = 7.11 <18.30, p = 0.0000416). In addition, most isolates in the two major subtypes appearing before August 17, 1998, belonged to the A2 group, while most isolates appearing after August 24, 1998, belonged to the A4 group (1.47 <RR = 9.82 <65.45, p = 0.0006). Finally, unlike A4, the earlier A2 subtype was not isolated in northern Greece.

### Conclusions

Our results indicate that PFGE is useful in distinguishing epidemiologically related *S.* Blockley isolates since two of the four nonoutbreak isolates displayed unique PFGE patterns, A7 and A8, while PFGE patterns A2 and A4 grouped most of the 29 outbreak isolates (11 and 12, respectively).

These two chromosomal fingerprints, differing by two DNA fragments, were associated with two distinct resistance phenotypes. The resistance phenotype of A4 isolates, ATCN, was identical to the earlier resistance phenotype of A2 isolates, ATC, except for the resistance to nalidixic acid. Nevertheless, these two PFGE/antibiotic resistance types, A2/ATC and A4/ATCN, displayed a clear distribution both in time and space.

The data may, therefore, indicate two main sources for the outbreak. Alternatively, and perhaps more likely, these two closely related types may together constitute the outbreak clone, evolved with time to acquire resistance to nalidixic acid. Resistance may well have originated in the food source, since several antibiotic classes are used as feed supplements in animal rearing and aquaculture in Greece: sulfonamides (trimethoprim/sulfathiazine), tetracyclines (oxytetracycline), and quinolones (oxolinic acid). However, as in other European countries (17,18), the epidemiologic investigation did not locate a common source to account for the wide geographic spread of cases (19). Although travel was not mentioned in the Greek patients' questionnaire responses, the possibility that the source was an imported food cannot be ruled out. The association with smoked eel of Italian origin in the German outbreak has not been microbiologically confirmed (17). The only other previous European report of a human outbreak attributed to *S.* Blockley, probably from vegetables contaminated by this organism, which was prevalent in irrigation water in the Spanish region of Granada, is anecdotal (13). A documented *S.* Blockley enteritis epidemic in a U.S. hospital in 1966 was attributed to contaminated ice cream; however, this was also not microbiologically confirmed (7).

While this serotype may remain important in Europe, its high rates of resistance to kanamycin, streptomycin, tetracycline, and chloramphenicol, which were in agreement with studies from the Far East (3) and Spain (12), are cause for concern. Unlike the Far Eastern strains, no resistance to ß-lactam antibiotics or cotrimoxazole was observed in our study. The two dominant resistant phenotypes of *S.* Blockley from natural polluted waters in Spain were sulfonamides, streptomycin, and tetracycline; and neomycin, streptomycin, kanamycin, tetracycline, and chloramphenicol (12), as in the Greek strains, except for the absence of resistance to nalidixic acid.

In agreement with differences in animal reservoirs and transmission routes and therefore the mechanism of resistance acquisition among different *Salmonella* serotypes, the main patterns of resistance observed in *S.* Blockley were distinct from those predominating in the two major serotypes from isolates of both human and animal food origin in Greece. In *S.* Enteritidis, the most frequent resistance phenotype was resistance to ampicillin (24), while in *S.* Typhimurium, the most frequent resistant phenotype was resistance to sulfonamides and streptomycin (A. Markogiannakis, P.T. Tassios, N.J. Legakis, unpub. obs.). Furthermore, the considerably high rate of resistance to nalidixic acid is equally unprecedented in both the Far Eastern and Spanish *S.* Blockley isolates and in other salmonella serotypes from Greece. Since resistance to nalidixic acid can be a precursor of resistance to fluoroquinolones, one of the two drug classes of choice for invasive salmonella disease, this feature of these *S.* Blockley strains is particularly disturbing. *S.* Blockley, previously a prevalent serotype in the Far East but rare elsewhere, nevertheless posed a public health problem in several European countries. The source of the European outbreaks, however, remains unclear. Given the increased international commerce in food, a collaborative study would be useful in identifying potential similarities between the recent European strains and established strains from the Far East.

### Acknowledgments

We thank Veneta Lukova and Soula Christou for assistance with antibiotic susceptibility testing.

This work was funded in part by the Ministry of Health and Welfare.

Dr. Legakis is professor and head of the department of Microbiology of the Medical School of the National University of Athens. His research interests include molecular typing, mechanisms of antibiotic resistance of bacterial pathogens, and molecular diagnosis of infections.

### References

1. Fisher IST, on behalf of Enter-net participants. Enter-net Quarterly Salmonella Report - 98/3 (1998 Jul-Sep). Available from: URL: http://www2.phls.co.uk/reports/latest.htm
2. Limawongpranee S, Hayashidani H, Okatani AT, Ono K, Hirota C, Kaneko K, et al. Prevalence and persistence of Salmonella in broiler chicken flocks. J Vet Med Sci 1999;61:255-9.
3. Matsushita S, Yamada S, Sekiguchi K, Kusunokui J, Ohta K, Kudoh Y. Serovar-distribution and drug-resistance of Salmonella strains isolated from domestic and imported cases in 1990-1994 in Tokyo. Kansenshogaku Zasshi 1996;70:42-50.
4. Yasin RM, Jegathesan MM, Tiew CC. Salmonella serotypes isolated in Malaysia over the ten-year period 1983-1992. Asia Pac J Public Health 1996-97;9:1-5.
5. Rusul G, Khair J, Radu S, Cheah CT, Yassin RM. Prevalence of Salmonella in broilers at retail outlets, processing plants and farms in Malaysia. Int J Food Microbiol 1996;33:183-94.
6. Sasipreeyajan J, Jerngklinchan J, Koowatananukul C, Saitanu K. Prevalence of salmonellae in broiler, layer and breeder flocks in Thailand. Trop Anim Health Prod 1996;28:174-80.
7. Morse LJ, Rubenstein AD. A food-borne institutional outbreak of enteritis due to *Salmonella blockley*. JAMA 1967;202:939-40.
8. Albert S, Weber B, Schafer V, Rosenthal P, Simonsohn M, Doerr HW. Six enteropathogens from a case of acute gastroenteritis. Infection 1990;18:381-2.
9. van Duijkeren E, Sloet van Oldruitenborgh-Oosterbaan MM, Houwers DJ, van Leeuwen WJ, Kalsbeek HC. Equine salmonellosis in a Dutch veterinary teaching hospital. Vet Rec 1994;135:248-50.
10. Mallaret MR, Turquand O, Blatier JF, Croize J, Gledel J, Micoud M, et al. Human salmonellosis and turtles in France. Rev Epidemiol Sante Publique 1990;38:71-5.
11. Levre E, Valentini P, Brunetti M, Sacchelli F. Stationary and migratory avifauna as reservoirs of *Salmonella*, *Yersinia* and *Campylobacter*. Ann Ig 1989;1:729-40.
12. Morinigo MA, Cornax R, Castro D, Jimenez-Notaro M, Romero P, Borrego JJ. Antibiotic resistance of *Salmonella* strains isolated from natural polluted waters. Journal of Applied Bacteriology 1990;68:297-302.
13. Garcia-Villanova Ruiz B, Cueto Espinar A, Bolanos Carmona MJ. A comparative study of strains of salmonella isolated from irrigation waters, vegetables and human infections. Epidemiol Infect 1987;98:271-6.
14. Matsushita S, Yamada S, Inaba M, Kusunoki J, Kudoh Y, Ohashi M. Serovar distribution and drug resistance of Salmonella isolated from imported and domestic cases in 1980-1989 in Tokyo. Kansenshogaku Zasshi 1992;66:327-39.
15. Bangtrakulobth A, Suthienkul O, Kitjakara A, Pornrungwong S, Siripanichgon K. First isolation of *Salmonella blockley* in Thailand. Southeast Asian J Trop Med Public Health 1994;25:688-92.

16. Atanassova V, Matthes S, Muhlbauer E, Helmuth R, Schroeter A, Ellendorff F. Plasmid profiles of different Salmonella serovars from poultry flocks in Germany. Berl Munch Tierarztl Wochenschr 1993;106:404-7.
17. Hamouda O. Outbreak of *Salmonella blockley* infections in Germany—preliminary investigation results. Eurosurveillance Weekly 1998;2:980924. Available at URL: http://www.eurosurv.org/1998/980924.htm
18. Benons L. *Salmonella blockley* infections in England and Wales, 1998. Eurosurveillance Weekly 1998;2:980924. Available at URL: http://www.eurosurv.org/1998/980924.htm
19. Vassilogiannakopoulos A, Tassios P, Lampiri M. *Salmonella blockley* infection in Greece. Eurosurveillance Weekly 1999;3:990408. Available at URL: http://www.eurosurv.org/1999/990408.htm
20. Kauffman F. Serological diagnosis of *Salmonella* species. Copenhagen (Denmark): Munksgaard; 1972.
21. National Committee for Clinical Laboratory Standards. Methods for dilution antimicrobial susceptibility tests for bacteria that grow aerobically. Approved Standard M7-A2. Wayne (PA): The Committee; 1997.
22. Tassios PT, Vatopoulos AC, Mainas E, Gennimata D, Papadakis J, Tsiftsoglou A, et al. Molecular analysis of ampicillin-resistant sporadic *Salmonella typhi* and *Salmonella paratyphi* B clinical isolates. Clin Microbiol Infect 1997;3:317-23.
23. Tenover FC, Arbeit RD, Goering RV, Mickelsen PA, Murray BE, Persing DH, et al. Interpreting chromosomal DNA restriction patterns produced by pulsed-field gel electrophoresis: criteria for bacterial strain typing. J Clin Microbiol 1995;33:2233-9.
24. Tassios PT, Markogiannakis A, Vatopoulos AC, Katsanikou E, Velonakis EN, Kourea-Kremastinou K, et al. Molecular epidemiology of antibiotic resistance of *Salmonella enteritidis* during a 7-year period in Greece. J Clin Microbiol 1997;35:1316-21.
